# Trypanocide Use and Molecular Characterization of Trypanosomes Resistant to Diminazene Aceturate in Cattle in Northern Côte D’Ivoire

**DOI:** 10.3390/tropicalmed9090192

**Published:** 2024-08-24

**Authors:** Jean-Yves Ekra, Eliakunda Michael Mafie, Henri Sonan, Michael Kanh, Biégo Guillaume Gragnon, Edouard K. N’Goran, Jagan Srinivasan

**Affiliations:** 1 Department of Veterinary Microbiology Parasitology and Biotechnology, Sokoine University of Agriculture, Morogoro 67125, Tanzania; eliakunda.mafie@sua.ac.tz; 2 SACIDS Africa Centre of Excellence for Infectious Diseases, SACIDS Foundation for One Health, Sokoine University of Agriculture, Morogoro 67125, Tanzania; 3 Department of Biology and Biotechnology, Worcester Polytechnic Institute, Worcester, MA 01609, USA; 4 Unité de Formation et de Recherche (UFR) des Sciences Biologiques, Département de Biochimie-Génétique, Université Peleforo Gon Coulibaly, Korhogo BP1328, Côte d’Ivoirekanhmichael@upgc.edu.ci (M.K.); 5 Laboratoire National d’Appui au Développement Agricole (LANADA), Korhogo BP1328, Côte d’Ivoire; lrk@lanada.ci

**Keywords:** trypanocide, resistance, trypanosome, cattle, Côte d’Ivoire

## Abstract

The resistance of trypanosomes to the doses of trypanocide administered by farmers to their animals acts as a real brake on efforts to control to combat African trypanosomiasis. Thus, in-depth knowledge of the use of these different molecules and their resistance profiles will be necessary to establish an integrated strategy to combat African trypanosomiasis. To achieve these objectives, a participatory survey among farmers and a resistance diagnosis of trypanosome strains identified in three regions of northern Côte d’Ivoire (Bagoué, Poro and Tchologo) was carried out using the PCR-RFLP technique, followed by sequencing of genes of interest. This study made it possible to identify three molecules that are commonly used by 85% (63/74) of farmers. In descending order of use, we identified Isometamidium chloride (43%), Diminazene aceturate (28%) and Homidium bromide (14%). Three species of trypanosomes, *Trypanosoma congolense*, *Trypanosoma. theileri* and *Trypanosoma vivax*, were identified in farms, and only one strain had the adenosine transporter gene (*Trypanosoma congolense*), but this strain was sensitive to the Diminazene aceturate molecule. Comparison of the sequence of this trypanosome strain showed that it is different to the Kenyan strain diagnosed as resistant to the Diminazene aceturate molecule. This study shows that a variety of trypanocides are used by farmers, and that the resistance profile of the strains to the Diminazene aceturate molecule could not be observed. However, it is important to further investigate the other molecules encountered in Côte d’Ivoire.

## 1. Background

Animal pathogenic trypanosomes affecting livestock represent a major constraint on agricultural development and have a negative economic impact in Africa. They cause enormous losses due to direct animal mortalities, incur untimely expenditure on medicines and lead to a drop in meat and milk production [1]. Faced with the consequences of trypanosomiasis for African populations and their livestock, many efforts at controlling the disease have been made in sub-Saharan countries. One example is the creation of PATTEC (the Pan African Tsetse and Trypanosomiasis Eradication Campaign). This project aimed to create zones definitively free of the tsetse fly and trypanosomiasis. Their actions focused on tsetse fly transmission vectors, to break the transmission cycle. Despite PATTEC’s efforts, the sub-Saharan region remains tsetse infested.

Faced with these losses and the persistence of animal trypanosomiasis, the only recourse available to livestock farmers is chemical control, notably trypanocides. Chemotherapy and chemoprophylaxis are the mainstay of animal trypanosomiasis control, ensuring animal health and production in enzootic countries [2]. It is estimated that more than 35 million doses of trypanocides are used annually in Africa [3]. Only six compounds are currently authorized [4]. These are Diminazen aceturate, Homidium bromide/chloride, Isometamidium chloride, Quinapyramine sulphate, Suramin sodium and Melarsomine dihydrochloride. By far the most widespread use is of two compounds, Diminazen aceturate and Isometamidium chloride, widely applied against animal trypanosomiasis in Africa [5,6]. However, there are a growing number of reports of resistance to this handful of existing chemicals, notably to Diminazene and Isometamidium. Indeed, the massive and somewhat abusive use of trypanocides has resulted in the emergence of resistance to these molecules [7]. Cases of chemoresistance have been reported in 21 African countries, notably in Côte d’Ivoire [2,8,9,10,11,12].

However, resistance to these molecules is not well known in Côte d’Ivoire, as most researchers have used in vivo and in vitro culture methods to confirm resistance, as shown by the work of Yao et al. in 2021 [12]. These methods are laborious and time-consuming [13], making them difficult to implement in endemic countries. With this in mind, molecular methods have been developed to identify resistant trypanosomes [10,14]. Diminazene aceturate has been extensively studied for resistance, largely due to its widespread use as a trypanocidal compound, as previously highlighted. In *Trypanosoma brucei brucei*, it is well established that the efficacy of Diminazene is closely tied to the expression of the purine-type P2 adenosine transporter, *TbAT1* [15,16,17]. This transporter plays a crucial role in the drug’s uptake into the parasite, making it a key factor in determining treatment success. However, *TbAT1* is not the sole pathway for Diminazene transport; the high-affinity pentamidine transporter (*HAPT1*) also contributes to drug uptake, albeit as a secondary route [18,19]. The link between *TbAT1* and resistance to Diminazene in *T. brucei* has been firmly established, with resistance often resulting from mutations that lead to the loss of function of this critical transporter [18]. This relationship underscores the importance of *TbAT1* as a determinant of drug sensitivity and resistance.

In a similar vein, Delespaux and colleagues proposed the resistance mechanism of *Trypanosoma congolense* to Diminazene [20]. Through a BLAST search of the *TbAT1* gene, a putative P2-type adenosine transporter gene in *T. congolense* was identified and named *TcoAT1*. This gene is considered an ortholog of *TbAT1* and has been implicated as a key factor in Diminazene resistance in *T. congolense*. The identification of *TcoAT1* as a resistance gene further emphasizes the critical role that adenosine transporters play in mediating the effectiveness of trypanocidal compounds like Diminazene aceturate.

Until now, there have been no studies carried out in Côte d’Ivoire using molecular biology methods to identify drug-resistant trypanosomes. Being able to identify drug-resistant trypanosomes in cattle using genetic engineering techniques would seem to be one of the best approaches to map drug resistance more easily, to solidly and quickly strengthen strategies to combat African animal trypanosomiasis. The objective of this work was to identify the different trypanocides used by farmers, to investigate the presence of resistant strains of trypanosomes and their distribution, and to carry out their molecular characterization.

## 2. Materials and Methods

### 2.1. Sample Collection and Interviews with Farmers

**Figure 1:**
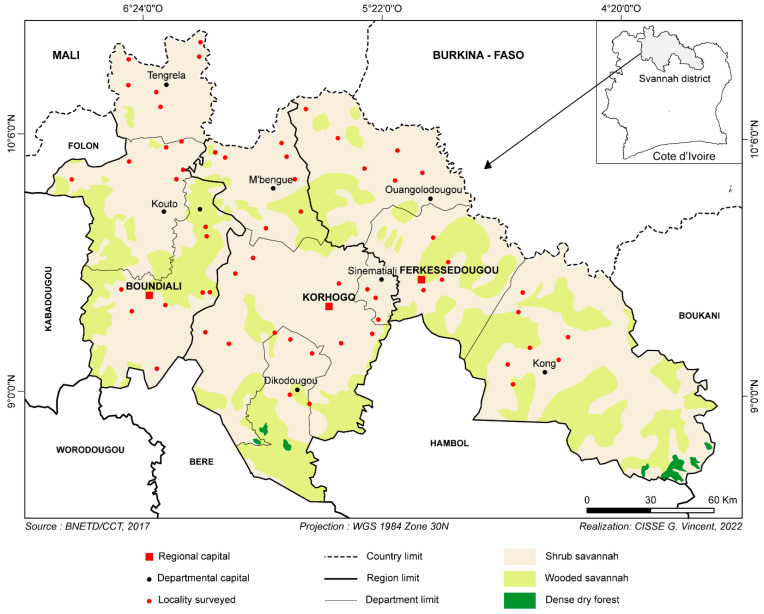
Study area.

This study was conducted in northern Côte d’Ivoire (Figure 1) from August to November 2021 in the savannah region, known as the most significant breeding area. To collect samples from the livestock of interest, an interview was organized with each farmer to obtain essential information for our study. Among the data collected, we focused on the farmer’s information (Table 1) and on information related to the use of trypanocides. By cross-referencing the information, we determined the following variables: (1) the frequency of trypanocide use, (2) the person administering the injection and (3) the name of the drug used, to identify the active ingredient. All this information was recorded on a form specific to each farm. After the interview, with the farmer’s consent, the animals were randomly selected for blood sampling. Consistent with previous studies [21], blood samples were collected using EDTA tubes. These tubes were transported on ice to the laboratory and stored at −20 °C for DNA extraction and PCRs for trypanosome identification.

### 2.2. Questionnaire Validation and the Study’s Limitations

To validate the questionnaire for our study, it was crucial to consider the linguistic and cultural specificities of our target population, made up of Peulh- and Senoufo-speaking farmers. We ensured that the language, terminology and concepts used in the questionnaire were fully understood by the respondents. To do this, we first carried out a pilot test of the questionnaire with a few members of the target population, which enabled us to gather valuable feedback. Based on this feedback, we made the necessary adjustments to improve the clarity and relevance of the questions. In addition, we used interpreters with a perfect command of Peulh and Senoufo to ensure that the concepts were correctly conveyed and that the answers collected truly reflected farmers’ practices and perceptions regarding the use of trypanocides.

This study has several limitations. Firstly, the sample size is limited to certain areas of northern Côte d’Ivoire, which may not fully represent the diversity of agricultural practices or the range of trypanosome strains present throughout the region. This limitation could affect the generalizability of the results. In addition, the accuracy of data on trypanocide use relies heavily on self-reported information from farmers, which may be subject to memory bias, under-reporting or inaccuracies, potentially introducing variability and affecting the conclusions drawn regarding trypanocide use in the region.

In addition, language and cultural barriers pose communication challenges, given that the study population includes Peulh- and Senoufo-speaking farmers. Even with the use of interpreters, subtle nuances can be lost, which can affect the reliability of the data collected. In addition, the study represents a snapshot in time, capturing data at a specific point in time, which may not reflect long-term trends in trypanosome resistance patterns or trypanocide use, nor the impact of interventions implemented after the study period. Furthermore, the study may not take full account of environmental variables such as climate, vector distribution or ecological changes, which could significantly influence trypanosome transmission dynamics and resistance development.

Finally, the availability and quality of veterinary services in the study area could also influence both the use of trypanocides and the management of trypanosomiasis, potentially skewing the results of the study if not sufficiently considered.

### 2.3. DNA Extraction, Trypanosome Identification and Sequencing of Trypanosome Transporter Genes

DNA was extracted from the blood samples according to the instructions of the manufacturer using a commercially available kit (Quick-DNA Miniprep Plus Kit; Zymo Research, Irvine, CA, USA). Two types of PCR were used to identify trypanosome species. The first PCR was performed by targeting the ITS1 region of the trypanosome genome with the pair of primers (ITS1 CF: 5′-CCGGAAGTTCACCGATATTG-3′ and ITS1 BR: 5′-TTGCTGCGTTCTTCAACGAA-3′) described in Njiru’s work in 2005 [22]. The second PCR for trypanosome identification targeted the genome region of trypanosomes composed of the partial 18S, ITS1, 5.8S, ITS2 and partial 28S rDNA genes. This PCR is performed in two steps (Nested PCR) with two pairs of primers (ITS1/ITS2 and ITS3/ITS4) [16]. The PCR mix and protocol of these two types of PCR identification were performed as described in our previous study [21]. PCR products were electrophoresed in 1.5% agarose (BioTools Inc, Japan) in (TBE) buffer and stained using safe view (1.4 µL for 140 mL of TBE) before being visualized under UV light. The expected fragment size for individual trypanosome species was shown in Supplementary Table S1 of our previous study [21].

All positive samples of *Trypanosoma congolense* and *Trypanosoma vivax* were subjected to PCR to amplify the transporter gene. Trypanosome transporter gene amplification was performed using specifics primers. The primers Ade2F (5′-TAATCAAAGCTGCCATGGATGAAG-3′) as the forward primer and Ade2R (5′-GATGACTAACAATATGCGGGCAAAG-3′) as the reverse of the adenosine transporter gene (TbNT10) were used. The PCR was carried in a 50 µL mixture containing 1× of *Taq* 5× Master mix, 0.2 µM of each primer and <1000 ng of Template DNA from the positive sample. The PCR cycles were as follows: 95 °C for 5 min; 40 cycles: 95 °C for 30 s, 50 °C for 50 s, 68 °C for 1 min; and 68 °C for 5 min. PCR products were electrophoresed in 1.5% agarose (BioTools Inc, Japan) in (TBE) buffer and stained using safe view (1.4 µL for 140 mL of TBE) before being visualized under UV light. The expected fragment size was 648 base pairs.

Trypanosoma strains resistant to Diminazene aceturate were investigated using PCR-RFLP, as described by Vitouley and Delespaux [14,20]. For this detection, a DNA fragment of the *Trypanosoma* adenosine transporter gene (TbNT10), which is positive for the PCR test with TbNT10 primers, was purified from a 1.5% agarose gel using a FastGene Gel/PCR extraction kit (NIPPON Genetics Co., Tokyo, Japan). One part of the PCR product purified was digested with *DpnII* (recognition sequence ^GATC) and *BclI* (recognition sequence T^GATCA) endonuclease under 37 °C overnight. Theoretical digestion of the 648 bp Ade2 amplicon with *BclI* should yield fragments of 354-256-38 bp and 610-38 bp for resistant and susceptible strains, respectively. For *DpnII*, we should have 400-248 bp for resistant strains and no digestion (648 bp) for sensitive strains. Digestion products were finally resolved and photographed on an agarose gel.

The positive PCR products from the two target regions (rRNA genes and TbNT10) in this study were submitted to the ETON Company for direct sequencing by the Sanger method. Following that, the resulting fragments were edited and aligned in BioEdit 7.2 software using the Cluster W program. The DDBJ/EMBL/GenBank databases contain the nucleotide sequences PP188049, PP188052 and PP188051 for rRNA genes of the three *T. congolense* strains identified and OR940900 for the adenosine transporter gene (TbNT10) reported in this work.

### 2.4. Statistical Analysis and Sequence Analyses

To gain an overview of the behavior and use of medicines in our study area, the variables were defined based on the information received from the farmers and were subjected to descriptive analysis. For a better understanding, the age of the farmers was divided into two groups. This grouping was made on a conventional basis according to Percheron Annick’s proposal, which divides age into five groups: 18–24 years, 25–34 years, 35–49 years, 50–64 years and 65 years and over [23]. In our case, our population is between 50 and 80 years old. We have therefore divided our population into two age groups: 50 years to 65 years and 65 years to 80.

Concerning the sequence, the forward and reverse nucleotide sequences of each sample obtained after sequencing were manually edited and used to generate a consensus sequence for each sample in BioEdit. These consensus sequences were aligned with the sequences obtained from the Genbank by using the CLUSTAL W multiple alignment program [24]. The neighbor-joining trees were created in MEGA7 for the various regions targeted by PCR [25]. Maximum likelihood (ML) analysis was used to estimate the evolutionary history using the Kimura 2-parameter model [26] for both genome regions of the trypanosome targeted. The Maximum Composite Likelihood (MCL) method was used to estimate pairwise distance. The approximate likelihood ratio test (aLRT), an alternative to non-parametric bootstrap estimate of branch support [27], was used to assess the probability of inferred branches.

## 3. Results and Discussion

### 3.1. Results

#### 3.1.1. Socio-Economic Characteristics of Farmers and General Prophylactic Practices on Visited Farms

The farms visited in this study are traditional and mainly of a sedentary type (66: 90.41%). In terms of breeds, these farms are made up of Bos-taurus (N’Dama: 10%; Baoule: 11%), Bos-Indicus (Zebu: 23%) and crosses between Bos-taurus and Bos Indicus (Mere: 56%), as reported in our previous study [21]. The owners of the farms visited in our study area are generally Senoufo (59: 80.82%) with a low education level. Some owners are of foreign origin, coming from neighboring countries such as Mali and Burkina Faso. They are between 50 and 80 years old. They are farmers (59: 80.89%) of cash crops such as cotton, cashew and mangoes and food crops such as rice, maize and groundnut. Livestock farming is generally relegated to the background for family emergencies such as deaths, weddings and cultural celebrations. The cattle are a family heritage obtained by inheritance and passed on from generation to generation. The herds are kept by Peulhs originally from Mali (64: 87.67%) and occasionally from Burkina Faso. These Peulhs, originally from neighboring countries, have a tradition of herding. These Peulhs, by experience, treat their animals without recourse to veterinary services. Trypanocides generally use intramuscular injections (56: 76.71%) and, on rare occasions, subcutaneous injections (Table 1).

**Table 1:** General view of visited farms.

Variables	Modality		Region			Chi-Squared Tests		
		Bagoué	Poro	Tchologo	Total	X	df	P
Farmer’s language	Peulh	1 (1.37%)	6 (8.22%)	7 (9.59%)	14 (19.18%)			
	Senoufo	24 (32.88%)	18 (24.66%)	17 (23.29%)	59 (80.82%)	5.78	2	0.055
Age	50–65 years	12 (16.44%)	11 (15.07%)	13 (17.80%)	36 (49.31%)			
	65–80 years	13 (17.81%)	13 (17.81%)	11 (15.07%)	37 (50.68%)	0.36	2	0.835
School	Yes	24 (32.88%)	14 (19.18%)	16 (21.92%)	54 (73.97%)			
	No	1 (1.37%)	10 (13.70%)	8 (10.95%)	19 (26.03%)	10.02	2	0.007
Main activity	Crop grower	25 (32.87%)	18 (24.66%)	17 (23.29%)	59 (80.82%)			
	Breeding	1 (1.37%)	6 (8.22%)	7 (9.59%)	14 (19.18)	5.76	2	0.050
Type of farm	Sedentary	25 (34.25%)	24 (32.88%)	17 (23.29%)	66 (90.41%)			
	Semi-transhumance	0	0	7 (9.59%)	7 (9.59%)	15.81	2	0.001
Bouvier nationality	Peulh—Burkina Faso	1 (1.37%)	0	5 (6.85%)	6 (8.22%)			
	Peulh—Mali	24 (32.88%)	21 (28.76%)	19 (26.03%)	64 (87.67%)			
	Senoufo—Autochtone	0	3 (4.11%)	0	3 (4.11%)	13.68	4	0.008
Injection mode	Intra-muscular	24 (32.87%)	16 (21.92%)	16 (21.92%)	56 (76.71%)			
	Subcutaneous	0	2 (2.74%)	4 (5.48%)	6 (8.22%)			
	No injection	1 (1.37%)	6 (8.22%)	4 (5.48%)	11 (15.07%)	9.63	4	0.047
Injector	Technician	8 (10.96%)	8 (10.96%)	10 (13.70%)	26 (35.62%)			
	Self-medication	17 (23.28%)	16 (21.92%)	14 (19.18%)	47 (64.38%)	0.58	2	0.758

#### 3.1.2. Livestock Farming and Use of Trypanocides

A total of 74 farmers were visited. They use various drugs against trypanosomes, namely, Veriben, Berenil, Ethidium bromide and Trypamidium-Samorin. Trypamidium-Samorin is the most widely used; 32 farmers (43%) use this drug. These drugs comprise three active ingredients: Diminazene aceturate, Homidium bromide and Isometamidium chloride (Table 2). Isometamidium chloride was the active ingredient most frequently encountered and used by almost half of the farmers (43%: 32 farmers), followed by Diminazene aceturate, used by 28% (21 farmers), and Homidium bromide, used by 14% (10 farmers). It should be noted that some of these farmers do not use any anti-trypanosome medication (15%: 11 farmers).

**Figure 2:**
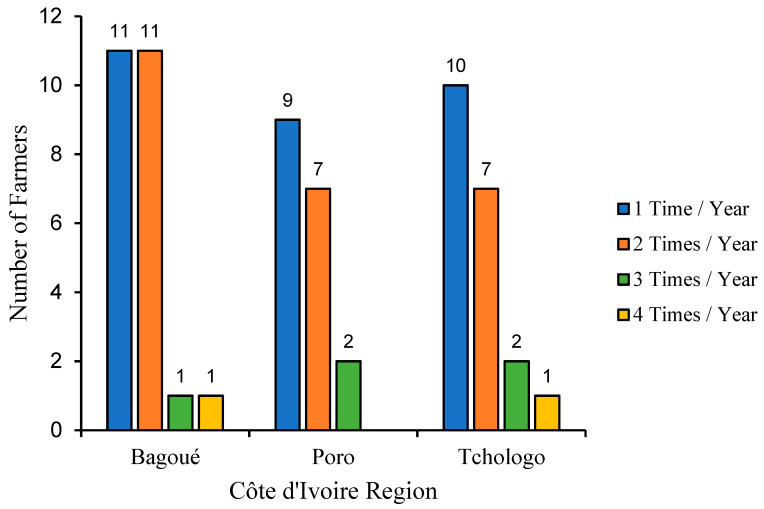
Frequency of use of trypanosome drugs in the Bagoué, Poro and Tchologo regions.

Figure 2 shows the frequency of use of these drugs over a year in the three regions of the present study area. In general, farmers in the three regions administrated these drugs to their animals between one and four times a year. We noted that most farmers treat their herds once a year, but the number of those who do so twice is not negligible. While the majority of farmers treat their herds one or two times a year, there are far fewer who treat their animals three or four times a year. Compared with the Poro and Tchologo regions, where most farmers treat their animals once a year, in the Bagoué region, the number of farmers who treat twice a year is in equal proportion to those who treat once.

#### 3.1.3. Trypanosome Species Identified on Farms

The PCR analyses carried out enabled us to identify three species of trypanosome circulating in the sampled farms. These are *T. vivax*, *Trypanosoma theileri* and *T. congolense*. Of the 74 farms, 37 farms (50%) were infested with these three species taken together. In what follows, we will focus on the farms that are infested, notably 50%.

**Figure 3:**
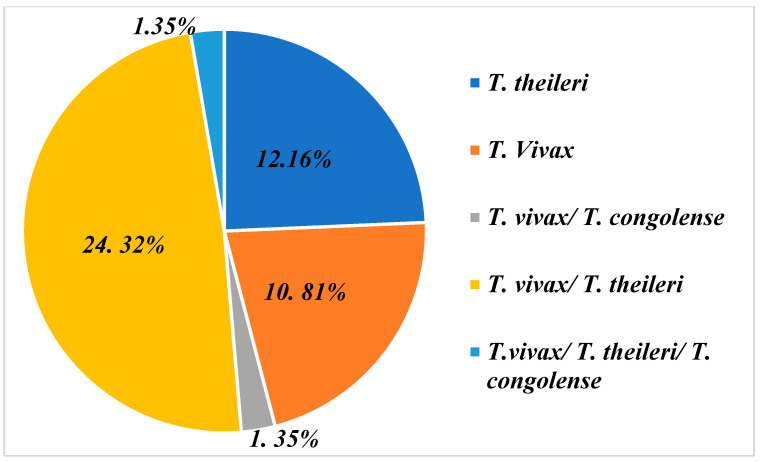
Infection and co-infection of trypanosome species found on farms representing 50% of farms sampled that presented with trypanosomes.

The graph in Figure 3 shows that farms are infested on the one hand either by a combination of different trypanosome species and on the other hand by one species only. In this diagram, we can see that the majority of farms are affected by a *T. vivax/T. theileri* co-infection, with a proportion of 24.32% [(18/74) (95% CI: 24.20%–24.44%)]. Then, we have farms infected with either *T. theileri* 12.16% [(9/74) (95% CI: 12.04%–12,28%)] or *T. vivax* 10.81% [(8/74) (95% CI: 10.69%–10.92%)]. Furthermore, a percentage of 1.35% [(1/74) (95% CI: 1.23%–1.47%)] was recorded for the combination of the three species, *T. vivax* /*T. theileri* and *T. congolense*.

#### 3.1.4. Molecular Identification of Diminazene Aceturate-Resistant Trypanosomes

**Figure 4:**
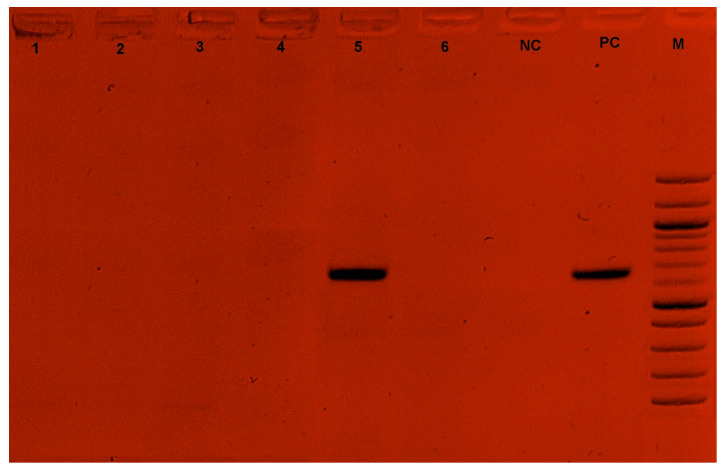
Gel electrophoresis of Ade2 PCR. 1,2,3,4,6: Negative sample; 5: Positive sample; NC: Negative control; PC: Positive control (648 bp~700 bp); M: Marker.

Among the 74 farmers surveyed, two had farms infected with *T. congolense*. Three strains of *T. congolense* (with a prevalence of 0.42%) were identified on these farms, as detailed in our previous study [21]. The adenosine transporter gene (TbNT10) was successfully amplified in one of the three *T. congolense* strains identified in this study. No amplifications were obtained for the *T. vivax* and *T. theileri* species (Figure 4).

**Figure 5:**
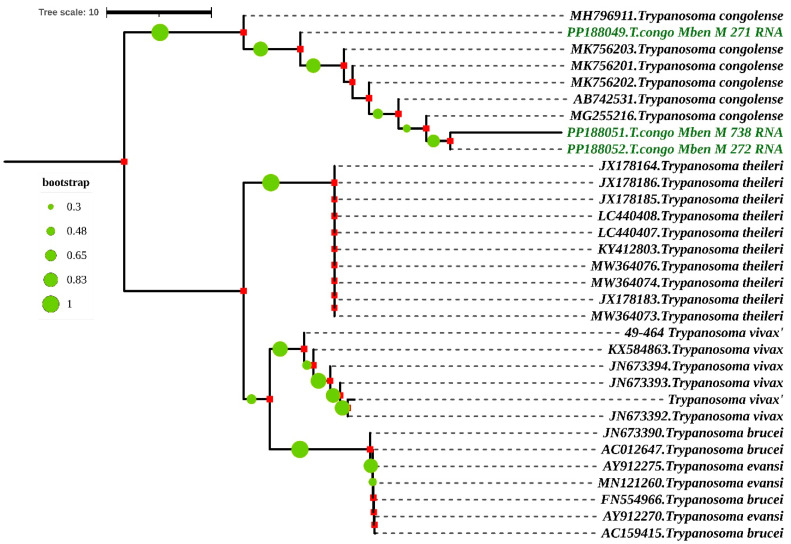
Phylogenetic analysis by Maximum Likelihood method based on Kimura 2-parameter model according to partial 18S, ITS1, 5.8S, ITS2 and partial 28S rRNA genes. The nucleotide sequences described in this study are written in green. Evolutionary history was inferred using the neighbor-joining method [28]. The percentage of replicate trees in which the associated taxa clustered together in the bootstrap test (1000 replicates) is shown next to the branches [29,30]. The evolutionary distances were computed using the Maximum Composite Likelihood method [31] and are in the units of the number of base substitutions per site. The rate variation among sites was modeled with a gamma distribution (shape parameter = 3). All ambiguous positions were removed for each sequence pair. Evolutionary analyses were conducted in MEGA7 [25] and iTOL (https://itol.embl.de/itol.cgi) accessed on 16 April 2024.

After purifying the 648 bp fragment corresponding to the TbNT10 gene, digestion of the purified fragment with *DpnII* and *BclI* enzymes revealed that none of the three *T. congolense* strains (0%) exhibited a profile consistent with a Diminazene aceturate-resistant *T. congolense* strain (Figure 5).

#### 3.1.5. Phylogenetic and Sequence Comparison

When the nucleotide sequences of the partial 18S, ITS1, 5.8S, ITS2 and partial 28S rRNA genes were compared, they shared a similarity of 79.26% to 91.57% with *T. congolense* species from Nigeria and Cameroon. The accession numbers of these two references sequences are MK756202.1 and MG255216. They were isolated, respectively, from the gut of *Glossina tachinoides* in Nigeria and from the proboscis of *Glossina morsitans submorsitans* in Cameroon (Figure 5).

**Figure 6:**
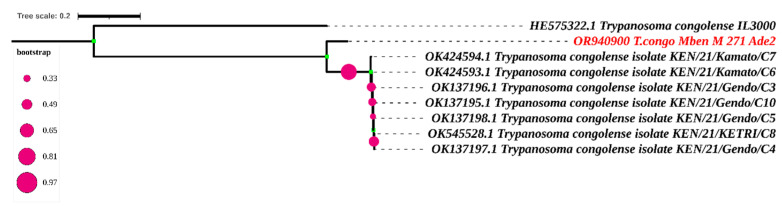
Phylogenetic analysis according to the putative adenosine transporter gene (Ade2) The nucleotide sequences described in this study are written in red. Evolutionary history was inferred using the neighbor-joining method [28]. The percentage of replicate trees in which the associated taxa clustered together in the bootstrap test (1000 replicates) is shown next to the branches [29,30]. The evolutionary distances were computed using the Maximum Composite Likelihood method [25] and are in the units of the number of base substitutions per site. The rate variation among sites was modeled with a gamma distribution (shape parameter = 3). All ambiguous positions were removed for each sequence pair. Evolutionary analyses were conducted in MEGA7 [25] and iTOL (https://itol.embl.de/itol.cgi) accessed on 16 April 2024.

**Figure 7:**
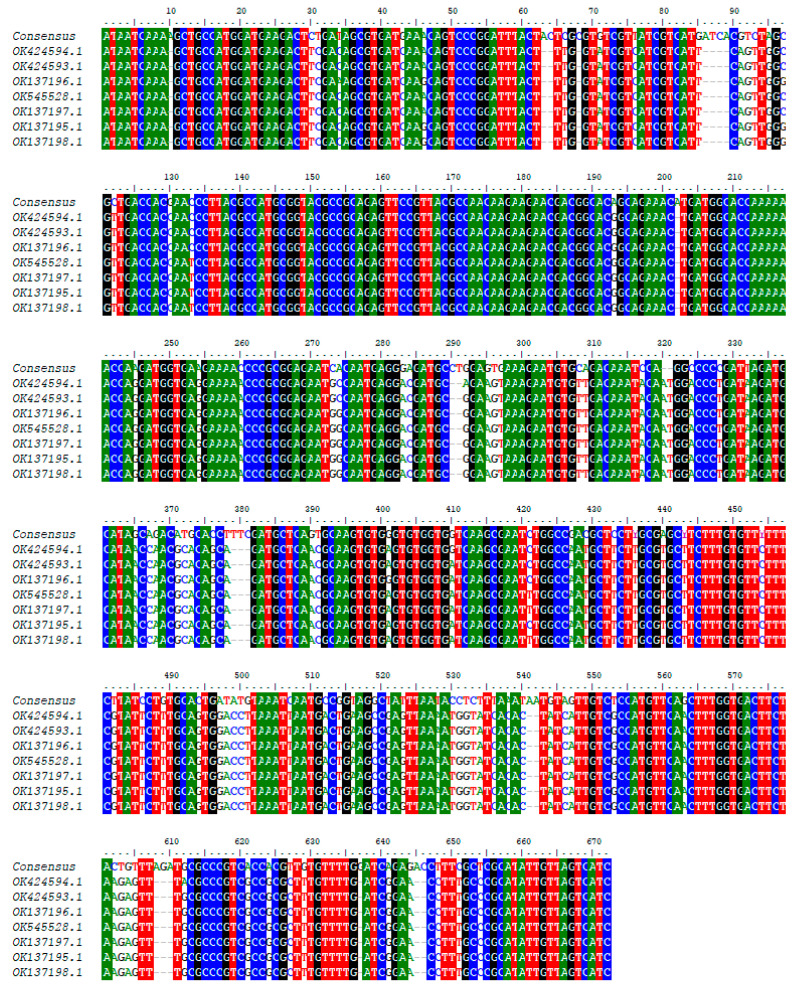
Nucleotide substitution observed based on adenosine transporter gene (TbNT10) sequence.

Only one sequence was obtained after sequencing the adenosine transporter gene (TbNT10). The blast results of this sequence enabled us to identify eight sequences similar to our own (Figure 6 and Figure 7). The adenosine transporter gene (TbNT10) nucleotide sequences of the present *T. congolense* were very similar to the deposited sequences of Kenya isolates of *T. congolense* (GenBank accession no. OK424594.1; OK424593.1; OK137196.1; OK545528.1; OK137197.1; OK137195.1; OK137198.1; and HE575322), with 80.27% to 80.92% identities. Regarding the nucleotide sequence of TbNT10 compared to these eight sequences, this similarity is with 111, 110, 111, 112, 112, 113 and 114 nucleotides substitutions, respectively, following the order listed above. As far as deletions (indel) are concerned, we were able to observe 22, as shown in Figure 7.

## 4. Discussion

Gathering data on the various trypanocides and the frequency of their administration could provide a better understanding of the current epidemiological situation of trypanosome resistance, to improve the control of African animal trypanosomiasis in these localities. Farmers use various drugs (Veriben, Berenil, Ethidium bromide and Trypamidium-Samorin). Farmers can obtain trypanocides from any street vendor who can boast of the merits of their products, leading to a diversity of products used by farmers. The lack of follow-up for farmers and the porous nature of the Ivorian borders means that some farmers closer to the borders use the services of certain agents from neighboring countries (Mali and Burkina Faso), who may supply products other than those approved in Côte d’Ivoire.

This work has shown that three major components (Diminazen aceturate, Homidium bromide and Isometamidium chloride) are used by farmers for trypanosome control, the most widely used being Isometamidium chloride. This observation is in line with those of researchers such as Tekle [13,31], who have observed the preponderant use of trypanocides based on Diminazen aceturate and Isometamidium, respectively, in Burkina Faso and Ethiopia. The major use of Isometamidium chloride would be due to the low availability of trypanocides based on Diminazen aceturate molecules on the Ivorian market, which would push breeders to turn to an alternative, particularly Isometamidium chloride. Indeed, due to recurrent complaints from farmers about the ineffectiveness of trypanocides based on Diminazen aceturate molecule, the structures in charge of sales have reduced their imports of trypanocides based on this molecule. This resistance to Diminazen aceturate was confirmed in studies by Yao et al. [12]. These studies showed that trypanosome species such as *T. vivax* resist this molecule. In fact, according to Toure [32], a single administration at a dose of 3.5–7 mg/kg for Diminazen aceturate or a dose of 0.25–0.8 mg/ kg for Isometamidium generally results in the recovery of animals infected by *T. vivax* and *T. congolense*. The protection afforded by these molecules can last for three to four months when Isometamidium is used at a dose of between 0.5 mg/kg and 0.8 mg/kg [32]. However, according to our discussions with farmers, we observe under-dosing and poor utilization of trypanocides. The effective concentration is not reached due to excessive dilution of trypanocide for economic reasons. With one sachet of trypanocide, farmers want to treat too many cattle whose weights they do not know, which leads to a reduced dose for each animal. Moreover, trypanocides are generally used once or twice a year in the Bagoué, Tchologo and Poro regions. This time interval is inappropriate for the drug’s prophylactic activity, resulting in loss of efficacy against certain strains of trypanosomes that acquire resistance. In addition, it is recommended that once a year, in addition to the commonly used molecules, the animals should be treated separately with another molecule to delay the development of resistance, according to the “sanative pair” concept [33]. Three (3) species are responsible for trypanosomiasis on the farms we visited (*T. vivax*, *T. theileri* and *T. congolense*). Mixed infections of *T. vivax* and *T. theileri* are the most common; however, it is important to note that *T. theileri* is not pathogenic, as mentioned by Desquesnes [34]. So, although it is abundant, it has no significant impact on animal health and therefore poses no risk in terms of lost production for farmers.

We have found the presence of the *T. congolense* species, whose virulence and resistance has been demonstrated by several researchers, notably Jamal, Simo and Delespaux [9,11,20]. The work of Desquesnes [2] has enabled us to link the acquisition of resistance in *T. congolense* to the Diminazen aceturate molecule to a mutation in the adenosine transporter gene. PCR-RFLP enables us to profile both resistant and non-resistant strains [2,14]. Our investigation of mutations in the adenosine transporter gene associated with resistance or sensitivity to the Diminazen aceturate molecule in *T. congolense* species enabled us to amplify this gene in only 1/3 of the *T. congolense* identified in this work. We had obtained a sensitive profile after digestion of the positive PCR product with the restriction enzymes *DpnII* and *BclI*, as described in the works of Vitouley and Delespaux [14,20]. These results are in line with those of Simo et al. [11], whose study was on the resistance of trypanosomes to the Diminazen aceturate molecule from tsetse in central Cameroon. They also obtained trypanosome strains with the adenosine transporter gene, but not all of these were resistant. This result may be due primarily to the low prevalence of the *T. congolense* strain obtained in this work. Also, most farmers used Isometamidium chloride and the farmers in whose animals this strain was observed used Homidium bromide. *T. congolense* resistance to these two molecules has not yet been investigated in terms of molecular diagnostics. This result is insufficient to draw any conclusions about the resistance or susceptibility of our trypanosome strains, especially as these strains have not been isolated for drug testing. Furthermore, this molecular method has only been confirmed for a single species, namely *T. congolense*. Currently, the most common species in sub-Saharan Africa is *T. vivax* [35,36,37].

In this context of the *T. congolense* resistance to Diminazene, some researchers believe that Diminazene resistance in *T. congolense* is not due to a reduction in transport capacity but is instead associated with a decrease in mitochondrial membrane potential [38]. Indeed, the hypothesis that resistance might result from reduced Diminazene uptake by the parasite was investigated by de Koning [16]. This work demonstrated that trypanosome strains expressing the adenosine transporter gene (*TbAT1*) in Trypanosoma brucei can absorb Diminazene. Conversely, trypanosomes with mutant adenosine transporter genes lost almost all Diminazene transport capacity. Based on this, the equivalent gene in *T. congolense*, known as *TcoTA1*, was identified as responsible for Diminazene uptake in this species [20]. Delespaux concluded that resistance in *T. congolense* to Diminazene is caused by a reduction in Diminazene transport capacity due to a point mutation in the *TcoAT1* gene [20]. However, this conclusion was challenged by Carruthers [38], who observed that folate inhibited the accumulation of DB75, a close analog of Diminazene, in the IL3000 strain of *T. congolense*, where Diminazene resistance was previously induced in vitro. He found that Diminazene uptake was slow, with low affinity, and was partly but reciprocally inhibited by folate [38]. The expression of folate transporters significantly sensitized the cells to both Diminazene and DB829, another close analog of Diminazene. Based on these results, Carruthers [38] proposed that Diminazene uptake in *T. congolense* occurs via multiple low-affinity mechanisms, including the aminopurine transporter P2 (*TcoAT1*) and folate transporters. However, genomic studies of Diminazene-resistant strains showed no significant changes in the sequence or expression of folate transporters. Nevertheless, all resistant clones displayed a moderate reduction in mitochondrial membrane potential (Ψm), leading to the conclusion that this resistance might be associated with a decrease in mitochondrial membrane potential Ψm.

Even if this is confirmed, it remains uncertain whether this is the primary cause of resistance, as transport capacity primarily concerns the plasma membrane or specific organelle membranes, while mitochondrial membrane potential is particular to the inner mitochondrial membrane. Following this line of reasoning, it would be prudent to demonstrate that Diminazene uptake requires energy-dependent transport mechanisms to enter the trypanosome. Therefore, a reduction in mitochondrial membrane potential could indirectly affect transport capacity by reducing the energy available for active transport processes.

The blast results of our unique adenosine transporter gene sequence yielded only eight sequences similar to the sequence in the NCBI database. Alignment using the Cluster W method, followed by phylogenetic analysis, revealed a difference with the strains from Kenya. Unlike HE575322, the other seven strains come from the same study by Okello [39]. They share the same substitution and deletion numbers when compared with our nucleotide sequence. The strong clade formed by these seven strains from Kenya within our sequence can be explained by the fact that they come from the same area. The small number of TbN10 sequences in the NCBI database reflects the amount of sequencing carried out on this gene. Most researchers stop after obtaining profiles after digesting the fragments with restriction enzymes, which reflects the performance and specificity of this diagnostic method.

## 5. Conclusions

In conclusion, this study identified that four trypanocide products, Veriben, Berenil, Ethidium bromide and Trypamidium-Samorin, are commonly used by farmers in northern Côte d’Ivoire to combat trypanosome infections. Among these, Diminazene aceturate and Isometamidium chloride emerged as the most frequently employed active ingredients. While the study identified trypanosome species such as *T. vivax*, *T. theileri* and *T. congolense*, none exhibited the Diminazene resistance genotype. However, one strain of *T. congolense* carried the transporter gene associated with resistance, and this strain closely resembles those found in Kenya. Although the hypothesis that *T. congolense* resistance to Diminazene is linked to a mutation in the transporter gene remains contentious, it is still considered the most plausible explanation.

Future research should focus on investigating further the resistance or sensitivity profiles of trypanosome species found in Côte d’Ivoire, especially concerning the other molecules identified in this study. Given the diverse active ingredients in commonly used trypanocides, it is crucial to develop a comprehensive strategy that employs multiple methods to study resistance more effectively and derive more accurate conclusions.

## Figures and Tables

**Table 2:** Summary of drug use by farmers.

Name of Drug	Active Substance	Chemical Structure	Percentage of Users
Veriben and Berenil	Diminazene aceturate	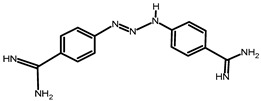	28% (21 farmers)
Ethidium bromide	Homidium bromide	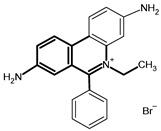	14% (10 farmers)
Trypamidium-Samorin	Isometamidium chloride	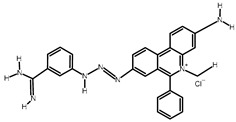	43% (32 farmers)
No Trypanocide	No active substance		15% (11 farmers)
			100% (74 farmers)

## Data Availability

All sequences generated in the present study are available in the GenBank database under the accession number mentioned in the text above.

## References

[1] Tesfaye D., Speybroeck N., de Deken R., Thys E. (2012). Economic Burden of Bovine Trypanosomosis in Three Villages of Metekel Zone, Northwest Ethiopia. Trop. Anim. Health Prod..

[2] Delespaux V., Dinka H., Masumu J., Van den Bossche P., Geerts S. (2008). Five-Fold Increase in *Trypanosoma congolense* Isolates Resistant to Diminazene Aceturate over a Seven-Year Period in Eastern Zambia. Drug Resist. Updates.

[3] Giordani F., Morrison L.J., Rowan T.G., De Koning H.P., Barrett M.P. (2016). The Animal Trypanosomiases and Their Chemotherapy: A Review. Parasitology.

[4] Venturelli A., Tagliazucchi L., Lima C., Venuti F., Malpezzi G., Magoulas G.E., Santarem N., Calogeropoulou T., Cordeiro-Da-silva A., Costi M.P. (2022). Current Treatments to Control African Trypanosomiasis and One Health Perspective. Microorganisms.

[5] Aregawi W.G., Gutema F., Tesfaye J., Sorsa A., Megersa B., Teshome P., Agga G.E., Ashenafi H. (2021). Efficacy of Diminazene Diaceturate and Isometamidium Chloride Hydrochloride for the Treatment of *Trypanosoma evansi* in Mice Model. J. Parasit. Dis..

[6] Ngumbi A.F., Silayo R.S. (2017). A Cross-Sectional Study on the Use and Misuse of Trypanocides in Selected Pastoral and Agropastoral Areas of Eastern and Northeastern Tanzania. Parasit. Vectors.

[7] Geerts S., Holmes P.H., Eisler M.C., Diall O. (2001). African Bovine Trypanosomiasis: The Problem of Drug Resistance. Trends Parasitol..

[8] Talaki E., Dao B., Kulo A.E., N’Feide T. (2013). Enquêtes Entomologique et Parasitologique Sur Les Trypanosomoses Bovines À La Station D’avétonou/Itra, Au Togo. Anim. Health Prod..

[9] Jamal S., Sigauque I., Macuamule C., Neves L., Penzhorn B.L., Marcotty T., Van Den Bossche P. (2005). The Susceptibility of *Trypanosoma congolense* Isolated in Zambézia Province, Mozambique, to Isometamidium Chloride, Diminazene Aceturate and Homidium Chloride. Onderstepoort J. Vet. Res..

[10] Mamoudou A., Zoli A., Tanenbe C., Andrikaye J.P., Bourdanne B., Marcotty T., Delespaux V., Clausen P.H., Geerts S. (2006). Assessment of the Resistance of Cattle Trypanosomes to Diminazene Aceturate and Isometamidium Chloride on the Adamawa Plateau in Cameroon Using a Field Test and a Test in Mice. Rev. DÉlevage Médecine Vét. Pays Trop..

[11] Simo G., Magang E.M.K., Mewamba E.M., Farikou O., Kamga R.M.N., Tume C., Solano P., Ravel S. (2020). Molecular Identification of Diminazene Aceturate Resistant Trypanosomes in Tsetse Flies from Yoko in the Centre Region of Cameroon and Its Epidemiological Implications. Parasite Epidemiol. Control.

[12] Yao L.S., Gragnon B.G., Komoin C.O., Bengaly Z., Komono B.D., Environnement D., Abrogoua U.N., Abidjan B.P. (2021). Chemoresistance of *Trypanosoma vivax* (Kinetoplastida: Trypanosomatidae). Int. J. Dev. Res..

[13] Tekle T., Terefe G., Cherenet T., Ashenafi H., Akoda K.G., Teko-Agbo A., Van Den Abbeele J., Gari G., Clausen P.H., Hoppenheit A. (2018). Aberrant Use and Poor Quality of Trypanocides: A Risk for Drug Resistance in South Western Ethiopia. BMC Vet. Res..

[14] Vitouley H.S., Mungube E.O., Allegye-Cudjoe E., Diall O., Bocoum Z., Diarra B., Randolph T.F., Bauer B., Clausen P.H., Geysen D. (2011). Improved Pcr-Rflp for the Detection of Diminazene Resistance in *Trypanosoma congolense* under Field Conditions Using Filter Papers for Sample Storage. PLoS Negl. Trop. Dis..

[15] Matovu E., Stewart M.L., Geiser F., Brun R., Mäser P., Wallace L.J., Burchmore R.J., Enyaru J.C., Barrett M.P., Kaminsky R. (2003). Mechanisms of arsenical and diamidine uptake and resistance in *Trypanosoma brucei*. Eukaryot. Cell.

[16] de Koning H.P., Anderson L.F., Stewart M., Burchmore R.J., Wallace L.J., Barrett M.P. (2004). The trypanocide diminazene aceturate is accumulated predominantly through the TbAT1 purine transporter: Additional insights on diamidine resistance in african trypanosomes. Antimicrob. Agents Chemother..

[17] Stewart M.L., Burchmore R.J.S., Clucas C., Hertz-Fowler C., Brooks K., Tait A., MacLeod A., Turner C.M.R., De Koning H.P., Wong P.E. (2010). Multiple Genetic Mechanisms Lead to Loss of Functional TbAT1 Expression in Drug-Resistant Trypanosomes. Eukaryot. Cell.

[18] Bridges D.J., Gould M.K., Nerima B., Mäser P., Burchmore R.J., De Koning H.P. (2007). Loss of the High-Affinity Pentamidine Transporter Is Responsible for High Levels of Cross-Resistance between Arsenical and Diamidine Drugs in African Trypanosomes. Mol. Pharmacol..

[19] Teka I.A., Kazibwe A.J., El-Sabbagh N., Al-Salabi M.I., Ward C.P., Eze A.A., Munday J.C., Mäser P., Matovu E., Barrett M.P. (2011). The Diamidine Diminazene Aceturate Is a Substrate for the High-Affinity Pentamidine Transporter: Implications for the Development of High Resistance Levels in Trypanosomes. Mol. Pharmacol..

[20] Delespaux V., Chitanga S., Geysen D., Goethals A., Van den Bossche P., Geerts S. (2006). SSCP Analysis of the P2 Purine Transporter TcoAT1 Gene of *Trypanosoma congolense* Leads to a Simple PCR-RFLP Test Allowing the Rapid Identification of Diminazene Resistant Stocks. Acta Trop..

[21] Ekra J.Y., N’goran E.K., Mboera L.E.G., Gragnon B.G., Assovié K.R.N., Mafie E.M. (2023). Molecular Epidemiological Survey of Pathogenic Trypanosomes in Naturally Infected Cattle in Northern Côte d’ivoire. Parasites Hosts Dis..

[22] Auty H., Anderson N.E., Picozzi K., Lembo T., Mubanga J., Hoare R., Fyumagwa R.D., Mable B., Hamill L., Cleaveland S. (2012). Trypanosome Diversity in Wildlife Species from the Serengeti and Luangwa Valley Ecosystems. PLoS Negl. Trop. Dis..

[23] Percheron A. (1988). Classes d’âge en question. Rev. Française Sci. Polit..

[24] Thompson J.D., Higgins D.G., Gibson T.J. (1994). Clustal W: Improving the Sensitivity of Progressive Multiple Sequence Alignment through Sequence Weighting, Position-Specific Gap Penalties and Weight Matrix Choice. Nucleic Acids Res..

[25] Kumar S., Stecher G., Tamura K. (2016). MEGA7: Molecular Evolutionary Genetics Analysis Version 7.0 for Bigger Datasets. Mol. Biol. Evol..

[26] Tamura K. (1992). Estimation of the Number of Nucleotide Substitutions When There Are Strong Transition-Transversion and G+C-Content Biases. Mol. Biol. Evol..

[27] Anisimova M., Gascuel O. (2006). Approximate Likelihood-Ratio Test for Branches: A Fast, Accurate, and Powerful Alternative. Syst. Biol..

[28] Saitou N., Nei M. (1987). The Neighbor-Joining Method: A New Method for Reconstructing Phylogenetic Trees. Mol. Biol. Evol..

[29] Felsenstein J. (1985). Confidence Limits on Phylogenies: An Approach Using the Bootstrap. Evolution.

[30] Tamura K., Nei M., Kumar S. (2004). Prospects for Inferring Very Large Phylogenies by Using the Neighbor-Joining Method. Proc. Natl. Acad. Sci. USA.

[31] Akoda K., Teko-Agbo A., Elh M.N., Walbadet L. (2008). Qualité des Médicaments vétérinaires en circulation au Sénégal. Article Original. RASPA.

[32] Toure M. (1973). Les Trypanocides et leur utilisation en medecine veterinaire. Rev. D’elevage Med. Vet. Pays Trop..

[33] Whitesides E.F. (1962). Interactions between Drugs, Trypanosomes and Cattle in the Field. Drugs, Parasites Hosts.

[34] Desquesnes M. (2017). Compendium of Diagnostic Protocols of the OIE Reference Laboratory for Animal Trypanosomoses of African Origin OIE Reference Laboratory for Animal Trypanosomoses of African Origin Compendium of Standard Diagnostic Protocols for.

[35] Kouadio I.K., Sokouri D., Koffi M., Konaté I., Ahouty B., Koffi A., Guetta S.P.N. (2014). Molecular Characterization and Prevalence of *Trypanosoma* Species in Cattle from a Northern Livestock Area in Côte d’Ivoire. Open J. Vet. Med..

[36] Acapovi-Yao G., Cisse B., Koumba C.R.Z., Mavoungou J.F. (2016). Infections Trypanosomiennes Chez Les Bovins Dans Des Élevages de Différents Départements En Côte d’Ivoire. Rev. Med. Vet..

[37] Ekra J.-Y., N’Goran E.K., Mboera L.E.G., Mafie E.M. (2023). Prevalence of Bovine Trypanosomiasis in Côte d’Ivoire: Systematic Review and Meta-Analysis. Onderstepoort J. Vet. Res..

[38] Carruthers L.V., Munday J.C., Ebiloma G.U., Steketee P., Jayaraman S., Campagnaro G.D., Ungogo M.A., Lemgruber L., Donachie A.M., Rowan T.G. (2021). Diminazene resistance in *Trypanosoma congolense* is not caused by reduced transport capacity but associated with reduced mitochondrial membrane potential. Mol. Microbiol..

[39] Okello I., Mafie E., Nzalawahe J., Eastwood G., Mboera L.E.G., Hakizimana J.N., Ogola K. (2023). *Trypanosoma congolense* Resistant to Trypanocidal Drugs Homidium and Diminazene and Their Molecular Characterization in Lambwe, Kenya. Acta Parasitol..

